# The effect of service robot occupational gender stereotypes on customers' willingness to use them

**DOI:** 10.3389/fpsyg.2022.985501

**Published:** 2022-11-02

**Authors:** Qian Hu, Xingguang Pan, Jia Luo, Yiduo Yu

**Affiliations:** ^1^School of Tourism Management, Chaohu University, Hefei, China; ^2^School of Business Administration, Lyceum of the Philippines University, Batangas City, Philippines; ^3^School of Business Administration, Faculty of Business Administration, Southwestern University of Finance and Economics, Chengdu, China; ^4^Business School, Chengdu University, Chengdu, China; ^5^Graduate School, Southwestern University of Finance and Economics, Chengdu, China

**Keywords:** service robot, gender stereotypes, performance expectancy, trust, willingness to use

## Abstract

Customers have obvious occupational gender stereotypes for service employees. In recent years, intelligent service robots have been widely used in the hospitality industry and have also been given gender characteristics to attract customers to use them. However, whether and when the usage of gendered service robots is effective remains to be explored. This research focuses on customers' occupational gender stereotypes and the gender of service robots, examining the influences of their consistency on customers' willingness to use service robots through three scenario studies. The findings suggest that: (1) The consistency between occupational gender stereotypes and service robot gender positively affects customers' willingness to use service robots. (2) Performance expectancy and trust are two psychological mechanisms underlying the above effect. (3) In the context of service failures, the consistency backfires and brings negative effects on willingness to use. This research extends the literature on customers' acceptance of anthropomorphized robots from the perspective of gender stereotypes and identifies the mechanisms behind the stereotype consistency effect. For practical implications, hotels should design and deploy gendered robots consistent with corresponding occupational gender stereotypes.

## Introduction

Throughout the whole service journey, customers contact hotel employees in various positions and correspondingly form different expectations regarding the different service posts. Among these expectations, gender might be the most salient one since it is the basic social symbol in the interpersonal interaction. Specifically, customers generally recognize that the receptionists should be more feminine, good at interpersonal skills, and behave in a kindly and lovely manner, while hotel managers should be more masculine with characteristics of vitality, rationality, and determination (Macrae et al., 1994; Fischer et al., 1997). Because of that, service employees who align with their occupational gender stereotypes are usually evaluated more favorably by customers (Mohr and Henson, 1996). Hotels therefore assign gender-consistent service personnel based on occupational gender stereotypes (White and White, 2006).

As smart technologies of robotics and artificial intelligence have developed recently, many robots have entered the service workplace and are gradually replacing human employees as independent service providers, since it has cost and efficiency advantages (Carpenter et al., 2009; Harris et al., 2018). For example, Hilton Hotel uses concierge robots to replace human concierges, and Aloft Hotel uses service robots to replace human attendants. Henn-na and FlyZoo are totally robot staffed hotels, where no human employees can be found at all. Given that occupational gender stereotypes are so deep-seated, technicians place gender characteristics upon service robots by utilizing voice, attire, and physical forms, in hope of maintaining and attracting customers (Eyssel and Hegel, 2012; Blut et al., 2021; Ahn et al., 2022).

Nonetheless, the existing literature remains well explored regarding whether and under what conditions service robot gender can increase customers' willingness to use this technology. Since the occupational gender stereotype is a critical aspect that customers use to associate the robot gender with service occupations, we propose that customers' perceived consistency between occupational gender stereotypes and robot gender would affect their service evaluations. High consistency brings a match with customers' fundamental cognitions in social interactions — perceived competence and warmth — which we refer to as the expectation for robots' competency to perform the service work. Therefore, customers' willingness to use service robots is significantly improved, and thus, we can trust the research.

The research grounds occupational gender stereotypes of social role theory and examines the impact of service robot gender on customers' willingness to use them. Three scenario studies were employed to test the relationship between service robot gender, occupational gender stereotype consistency, and customers' willingness to use, as well as the underlying mechanisms. By doing so, this paper theoretically contributes to the literature on the acceptance of service robots and provides practical implications for hospitality industries.

## Theoretical background and hypotheses development

### Service robot gender and consumers' acceptance

Service robots are system-based autonomous and adaptable interfaces that interact, communicate, and deliver service to an organization's customers (Wirtz et al., 2018). As technology advances, service robots obtain premium capabilities of data processing and analysis, which enables them to interact with consumers better (Marinova et al., 2017). Given this, service robots are normally seen as an innovative application to enhance service process and quality, and are gradually becoming an alternative to human employees in many areas of the service industry (Van Doorn et al., 2017). In the hospitality context, frontline employees in different positions contact customers frequently (Lv et al., 2022). Wirtz et al. (2018) defined robots providing services on the frontline as interactive robots, which are autonomous, flexible, and capable of interacting with and completing service tasks for customers. For hotels, service robots are becoming widely used since there is a great advantage in reducing labor costs and improving efficiency (Tuomi et al., 2021). On the other hand, the acceptance by customers cannot be ignored considering that it determines how long service robots remain in use and whether they can even completely replace human employees someday in hospitality. Early literature on customers' acceptance of service robots was mostly based on the traditional framework of technology acceptance theory, such as the technology acceptance model (TAM) and unified theory of acceptance and use of technology (UTAUT), to examine the impacts of technical features on customers' willingness to adopt (Fritz et al., 2016; Sundar et al., 2016). Gursoy et al. (2019) later suggested that compared to traditional technologies, AI devices (i.e., service robots) were characterized by higher-level intelligence and able to fill multiple roles in service contexts, thereby reducing the explainable power of the tenets of the above theories.

To address this issue, Gursoy et al. (2019) proposed the AI device use acceptance (AIDUA) model and further stated the roles of social influences, hedonic motivation, anthropomorphism, performance expectancy, effort expectancy, and emotion on service AI acceptance. Among them, anthropomorphism is the distinctive element that can distinguish AIDUA from traditional technology acceptance theories and thus has been well-concerned in the research area of service robots (Destephe et al., 2015; Wirtz et al., 2018). Prior research has suggested that physical features (i.e., head, face, and body) and non-physical features (i.e., making eye contact, using gestures, movement, and voice) can affect the extent to which service robots are anthropomorphized (Blut et al., 2021). As a fundamental facet of anthropomorphism, gender often serves as the salient observable cue of human likeness.

In fact, robots do not have a specific gender as they are made of metal, plastic, and silicon. Nevertheless, they are created for human environments and represent many social roles, such as assistants, friends, and even enemies (Dautenhahn et al., 2005). Therefore, people are inclined to consider and treat robots with a bipolar gender mindset. Considering this inclination, designers ascribe genders to robots through voice, attire, physical bodies, etc. (Eyssel and Hegel, 2012; Blut et al., 2021; Ahn et al., 2022), to enhance customers' acceptance.

Meanwhile, researchers have begun a primary investigation into the impact of robot gender on customers' acceptance. For example, Carpenter et al. (2009) conducted a survey and found that in the family environment, users were more likely to use female robots. Tay et al. (2013) suggested that customers preferred male robots in terms of robotic security guards. However, the service context is complex and volatile, and it is not clear whether male or female robots work better in gaining customers' acceptance. But one thing is at least certain, that increasing customers' willingness to accept is highly necessary to improve service robots' application. The question thus arises: in hospitality settings, can the gender of service robots enhance customers' willingness to use them? Our research aims to answer this and examines which gender, the male- or female-presenting service robot, is better for customers' usage.

### Occupational gender stereotype

The belief of occupational gender stereotypes is held relatively fixed by people (Fiske, 2018), which claims that men and women have different characteristics, and are suitable for different types of occupations (White and White, 2006). Social role theory proposes that the division of labor is based on gender, and gender stereotypes are rooted in the different social roles assigned to men and women (Eagly and Steffen, 1984). The belief in gender further leads to gender stereotypes in people: women are considered nurturing and socially oriented (communal), while men are considered competitive and performance-oriented (agentic) (Eagly and Johnson, 1990). Occupations with more professional features, inclined to a high level of competence, rationality, and assertiveness are referred to as male occupations; occupations inclined to be interdependent, passive, nurturing, and requiring interpersonal warmth are considered female occupations (Shinar, 1975). For example, women more often engage in occupations resembling the housewife role (such as teachers or nurses). Conversely, men mostly play the roles of going to work and paying the bills, and display confidence and leadership, which are considered the higher level of the occupational hierarchy (Vogel et al., 2003). Such division of labor matches the social expectations of men's and women's attributes with their roles (Eagly and Steffen, 1984). When women engage in masculine occupations or men engage in feminine occupations, people expect that some behaviors inconsistent with their gender roles will occur, and therefore, the perception of a mismatch with their “social roles” is produced, resulting in negative evaluations of their work (Eagly and Steffen, 1984).

As an important concept in the field of social psychology, occupational gender stereotypes have been widely considered. Literature on organizational behavior and management psychology has extensively examined the impact of occupational gender stereotypes on workplace bias, job satisfaction, and job preference. Relevant studies show that when women engage in occupations standing in stark contrast to their feminine stereotypes, their job satisfaction decreases, and the turnover rate increases (Rudman and Phelan, 2008). Similar conclusions apply to men when it comes to inconsistency. Therefore, both men and women usually choose occupations that keep the consistency of gender stereotypes (Gadassi and Gati, 2009). Research into consumer behavior regarding service marketing focuses on the impact of occupational gender stereotypes on service quality perception by customers and reaches the consensus that when occupational gender stereotypes are consistent with service providers' gender, customers evaluate service quality better (Mohr and Henson, 1996; Fischer et al., 1997; Pinar et al., 2017). For example, Mohr and Henson (1996) chose mechanics, nurses, and teachers as the target occupations, and revealed that perceived service quality was significantly lower when service provider gender mismatched with occupational gender stereotypes. Pinar et al. (2017) suggested that customers' occupational gender stereotypes for service providers would be affected by different cultural backgrounds, but no matter what the cultural background was, consistency always produced a higher perception of service quality. Though existing research has well-examined the direct influence of consistency of occupational gender stereotypes, the psychological “black box” behind it remains unknown, preventing a thorough understanding of this issue.

As intelligent technology develops, essentially gender-neutral robots are equipped with artificial gender traits and will gradually become the new service providers. Against such a background, does the positive outcome resulting from the consistency between occupational gender stereotypes and service providers' gender disappear due to the instrumental nature of robots? Does consistency still improve customers' willingness to use? If the answer is yes, what is the underlying psychological mechanism? All of these questions need to be further examined and discussed.

### Influence of consistency between occupational gender stereotypes and service robot gender on willingness to use

The computers are social actors (CASA) paradigm states that humans unconsciously perceive machines (i.e., computers, TVs, virtual assistants, etc.) as alive social actors and apply interpersonal relationship norms when interacting with these non-humanoid entities (Reeves and Nass, 1996). The paradigm of the interaction between humans and machines can be understood from two levels. At the first level, as social characteristics of machines appear, people's social needs get the response; When humans accept the interaction with machines, they change their previous attitude of treating machines as traditional tools, and unconsciously start to project interpersonal elements such as gender and intention onto machines, which refer to the second-level responses (Fiore et al., 2013; Tay et al., 2014; Seo, 2022). Therefore, when service robots endowed with gender characteristics by designers interact socially with customers in service settings, they are easily regarded as real service providers with distinctive genders rather than merely cold machines with different technical designs.

Stereotypes are a handy cognitive tool for humans that can be triggered and inspired merely by simple cues (Macrae et al., 1994). According to the CASA paradigm, when customers face robots in specific service positions, their implicit occupational gender stereotypes are activated. When a position requires rationality, vitality, and other so-called masculine characteristics, it is often considered a male occupation, and thus male robots should be employed (Tay et al., 2013). Under such circumstances, if the hotel sets a male robot in the position, there is a match with customers' occupational gender stereotypes, and resultingly customers are more willing to use service robots. In a similar vein, when customers face a position in line with warmth, emotionality, and other feminine characteristics, such a position is generally regarded as a female occupation, and it would be better to be conducted by female employees (Tay et al., 2014). If the hotel sets up female robots for this position, it is following occupational gender stereotypes, and correspondingly customers will have a higher willingness to use them. Briefly, the consistency between occupational gender stereotypes and service robot gender positively affects customers' willingness to use them; otherwise, it backfires. Therefore, we propose the following hypothesis:

*H1: Compared with the inconsistency between occupational gender stereotypes and service robot gender, consistency produces a higher willingness to use service robots by customers*.

### The mediating roles of performance expectancy and trust

According to stereotype content model (SCM), there are two fundamental cognitive dimensions in social interaction—competence and warmth (Cuddy et al., 2008). Presumably, in the context of human-machine interaction, consumers will naturally evaluate service robots from the perspective of performance skills and personal emotion. Based on the AIDUA model, performance expectancy plays a key role in human-machine interaction and directly affects a customers' willingness to use it (Gursoy et al., 2019). Additionally, according to interpersonal trust theories, trust is conducive to understanding human interaction with AI (Gillath et al., 2021). Thus, we chose performance expectancy and trust as mediating variables in our model from the above two dimensions. Performance expectancy is defined as the extent to which an individual believes that technology use helps improve job performance (Venkatesh et al., 2003). In the context of service robots, this concept refers to the extent to which customers think that service robots can complete the service process well (Horstmann and Krämer, 2019; Yang et al., 2022). When the technology matches with tasks that customers perform, customers believe that technology can help them carry out tasks well and have a higher perceived performance of the technology (Goodhue and Thompson, 1995), thereby improving performance expectations (Dishaw and Strong, 1999). According to occupational gender stereotypes, male occupations involve masculine traits such as rationality and vitality, while female occupations involve feminine traits such as warmth and tenderness. Therefore, when service robots show up with corresponding gender characteristics, customers expect them to be able to fulfill the requirements of the occupation, thus generating high-performance expectancy. Alternately, gender mismatch leads to low-performance expectancy. Performance expectancy plays an important role in affecting behavioral intention. A high level of performance expectancy means that the service robot can efficiently solve various problems and complete the service, and customers are also more willing to use the service robot (Venkatesh et al., 2003; Šumak and Šorgo, 2016; Hoque and Sorwar, 2017; Lv et al., 2022). Therefore, keeping the gender of service robots consistent with occupational gender stereotypes improves customers' performance expectancy on service robots, and further enhances their willingness to use them.

Trust refers to the attitude of customers toward the agents who help them achieve personal goals under uncertain and risky conditions (Lee and See, 2004). Compared with human employees, when consumers face service robots, they are already in an uncertain and risky situation, and individuals show different attitudes as they are served by different service robots, thereby affecting their trust level. When service robots appear with corresponding gender characteristics, consumers are more willing to believe that they can complete the work with ease, and their inner guard will be appropriately lowered, thereby strengthening personal trust. On the contrary, if the service robot's perceived gender is mismatched with the occupational stereotyped gender, consumers are more inclined to express doubts about whether the service robot can complete the work, which intensifies their inner uncertainty and reduces their trust. As a strong factor in affecting technology use (Gefen et al., 2003), the more customers trust the technology, the more likely they are to rely on it (Lee and Moray, 1992; Lee and See, 2004; Gallimore et al., 2019; Miller et al., 2021). In other words, customer trust is positively related to their dependence on service robots, and further positively related to their willingness to use them. Therefore, the consistency between service robot gender and occupational stereotypes of corresponding positions improves customers' trust in service robots, which ultimately enhances customers' willingness to use them.

Given all that, we propose the following hypotheses:

*H2: Performance expectancy plays a mediating role in the effect of the consistency between occupational stereotypes and service robot gender on customers' willingness to use them*.
*H3: Customer trust plays a mediating role in the effect of the consistency between occupational gender stereotypes and service robot gender on customers' willingness to use them*.

### The moderating role of service failure

The service outcomes exceeding customer expectations bring service success. A service failure occurs when customers' benefits suffer, and service providers fail to meet the customers' expectations. Customers therefore feel dissatisfied and even abandon the service firms (Lv et al., 2020).

As previously discussed, the gender of service robots consistent with the specific occupation fits the stereotype of customers and further facilitates social interactions, because customers expect that consistency guarantees performance and trust. However, service failures attributed to the robot whose gender is consistent with their occupation, elicit the expectancy disconfirmation, which can increase the dissatisfaction of customers (Choi et al., 2021). Scholars in the information processing literature have found a similar effect when people receive messages contradicting prior ideas (Herr et al., 1983). In addition, customers who perceive consistency in gender stereotypes are more likely to feel disappointed and even betrayed, in that they have already developed a positive association with these intelligent service providers (Grégoire and Fisher, 2008). Consequently, their service evaluation becomes worse than those who meet with inconsistent gendered robots. Briefly, in the context of service failures, customers encountering the service robot whose gender is consistent with the occupational gender stereotypes would feel that the robot could have done a better job and thus are dissatisfied. Accordingly, customers have lower performance expectancy and trust toward the service robot. We propose the following hypotheses:

*H4: Service failure moderates the effect of the consistency between occupational gender stereotypes and service robot gender on performance expectancy. Consistency has a positive effect on performance expectancy when services succeed. Consistency harms performance expectancy when services fail*.
*H5: Service failure moderates the effect of the consistency between occupational gender stereotypes and service robot gender on performance expectancy. Consistency has a positive effect on trust when services succeed. Consistency has a negative effect on trust when services fail*.

**Figure 1:**
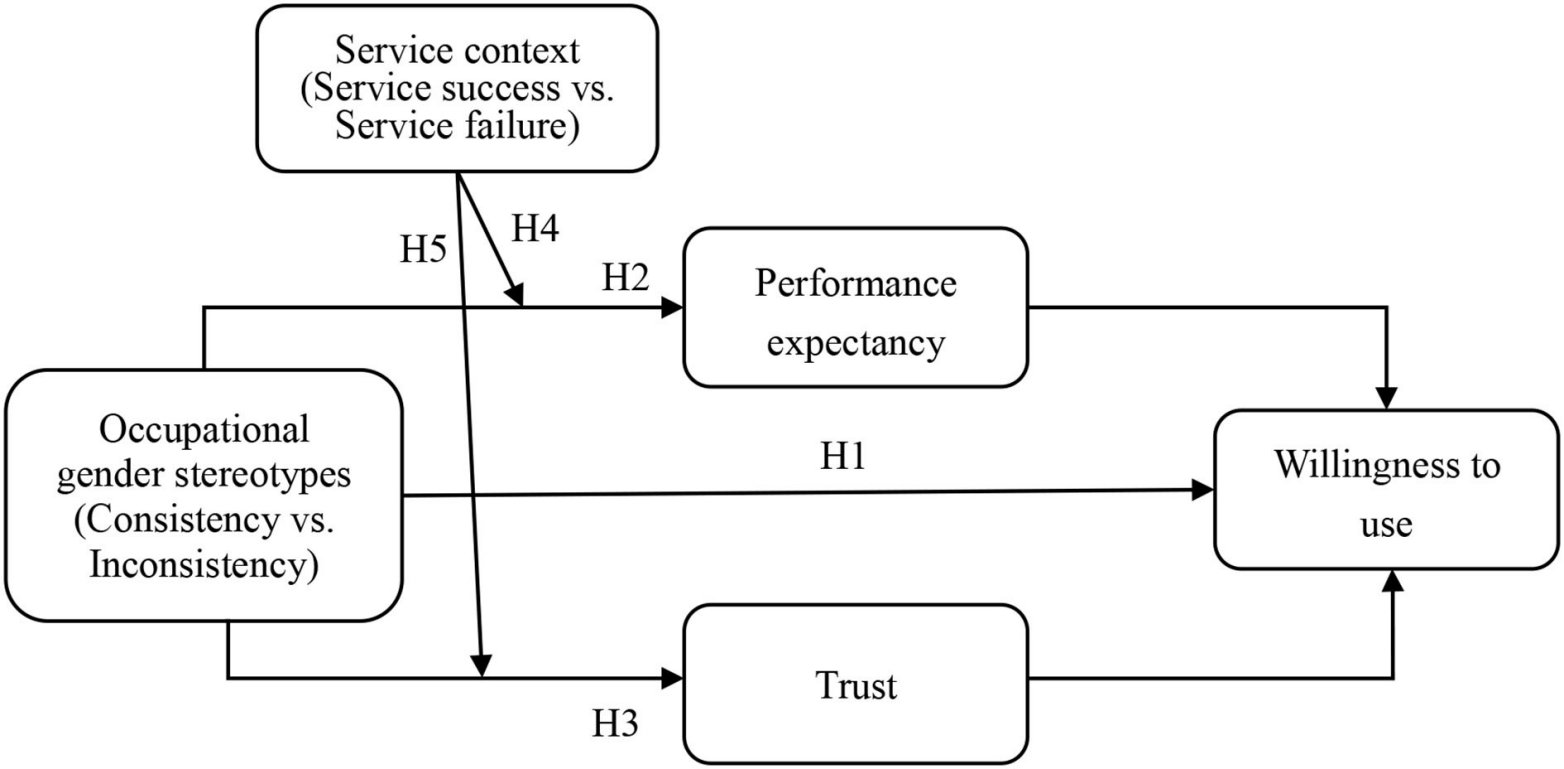
Research model.

The research model see Figure 1.

## Methodology

### Study 1

#### Research design

Study 1 examined the effect of consistency between occupational gender stereotypes and service robot gender on customers' willingness to use service robots by using the hotel reception scenario. A single-factor (Stereotypes: consistency vs. inconsistency) between-subject design was employed.

##### Pretest

Before the formal experiment, we conducted two pretests to guarantee the manipulation of occupational gender stereotypes and service robot gender, respectively. Fifty participants (54.0% Female; *M*_age_ = 28.86, SD = 2.98) participated in pretest 1A. One single item was used to measure occupational gender stereotypes of hotel receptionists, adapted from White et al. (1989): “Which gender do you think is more suitable for this occupation?” (rated on a 7-point scale from 1 = very suitable for a female to 7 = very suitable for male). The result of *T*-test demonstrated that participants' perception of gender stereotypes of hotel receptionists was significantly lower than median 4 (*M*_Stereotype_ = 2.60, *t*_(49)_ = −6.930, *p* < 0.001), suggesting that the perception of gender of hotel receptionists tends to be female.

Second, we tested the experimental materials with pretest 1B. We designed two virtual service robots based on the image of hotel receptionists. To manipulate the gender, we created soft features on the female service robot who wore a silk scarf, while we created hard features on the male service robot who wore a tie. Except for these differences, the two groups remained the same (such as standing posture, and reception posture, see Appendix 1). A total of 60 participants (46.7% Female; *M*_age_ = 28.70, SD = 2.98) were paid and recruited for Pretest 1B and were randomly assigned to the group of male service robots or female service robots. Participants were asked to identify the gender of the service robot and answered, “which gender do you think is more appropriate for the following image of the server?” (0 = Female, 1 = Male). The results indicated that in the female service robot group, 29 participants correctly identified the gender, and one participant incorrectly identified the gender; in the male service robot group, 23 participants correctly identified gender, and seven participants did not. The Chi-square test showed that there were significant differences between the groups (*p* < 0.001). Therefore, the materials can be used for further experiments.

##### Procedure and participants

**Figure 2:**
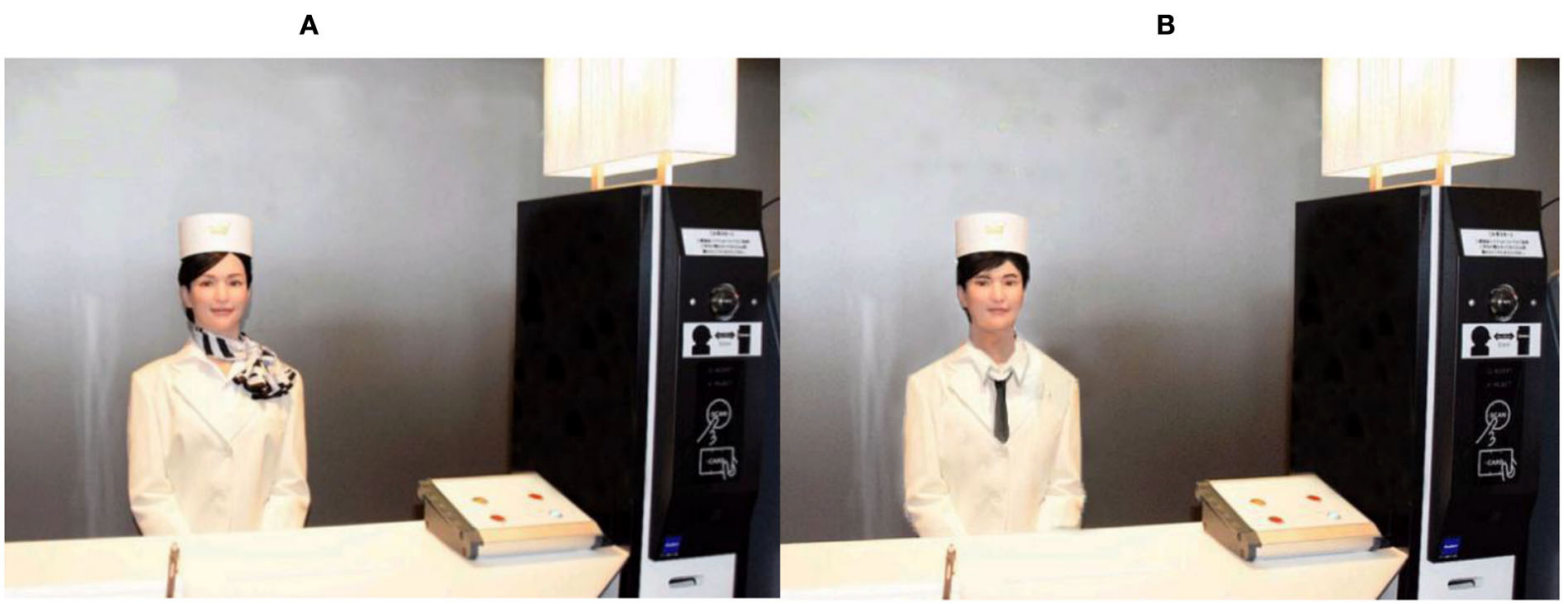
**(A)** Consistency group. **(B)** Inconsistency group.

Ninety volunteers (54.4% Female, *M*_age_ = 29.20, SD = 2.33) were paid and recruited for the formal experiment. First, participants were asked to read a service scenario of service robots working at the reception desk (see Appendix 2) and watch the images of service robots (Figure 2). Then, participants completed the questionnaire which consisted of two parts. In the first part, we measured the occupational gender stereotypes, gender perception of service robots, and willingness to use service robots. The measurement of occupational gender stereotypes and gender perception of service robots were the same as the two pretests. The willingness to use a service robot was measured by three items adapted from Agag and El-Masry (2016), and the items were appropriately modified and adjusted according to the experimental scenario: “I look forward to using this service robot”; “I prefer to use this service robot”; “I would like to use this service robot” (rated on a 7-point scale, 1 = Strongly disagree, 7 = Strongly agree). The second part asked for the demographic information of the participants (gender, age, etc.).

#### Results

##### Manipulation check

The results of *T*-test showed that the gender stereotypes perception of the hotel reception robots was significantly lower than the median of 4 (*M*_Stereotype_ = 2.96, *t*_(89)_ = −5.51, *p* < 0.001). In the consistency group, 40 participants correctly identified the service robot as female and four participants incorrectly identified the service robot as male. Similarly, in the inconsistency group, 38 participants correctly identified the service robot as male, and eight participants incorrectly identified the service robot as female. We excluded the participants who incorrectly judged the gender of the service robots and finally obtained 78 valid samples (*N*_consistency_ = 40, *N*_inconsistency_ = 38; 51.2% Female; *M*_age_ = 27.30, SD = 3.01).

##### Dependent variable

One-way ANOVA showed that the willingness to use the service robot (α = 0.803) was significantly higher in the consistent group than in the inconsistency group (*M*_consistency_ = 5.81, SD = 0.72; *M*_inconsistency_ = 5.33, SD = 1.15; *F*_(1, 76)_ = 4.83, *p* = 0.031, η^2^ = 0.060). Therefore, H1 was supported.

#### Discussion

In Study 1, we tested the impact of the consistency between occupational gender stereotypes and service robot gender on customers' willingness to use. The results indicated that customers are more willing to use the service robot when occupational gender stereotypes were consistent with the robot's gender compared to when they were inconsistent.

When interacting with customers, service robots' gender differences are not only reflected in their visual images, but also in voice. In addition, Study 1 only tested that customers' willingness to use service robots was affected by the consistency between gender and occupational stereotypes. The psychological mechanism behind is still unknown. Therefore, Study 2 would manipulate the experimental materials with voice and examine the main effect and the mediating mechanism.

### Study 2

#### Research design

Study 2 had three objectives. First, Study 1 chose the occupation of the hotel receptionist that reflects femininity. To increase the robustness, Study 2 employed a male-dominated occupation, hotel bellmen scenario to retest the effects. Second, Study 1 manipulated the gender of the service robot by using a visual image. Voice is also an important form of human-computer interaction that helps identify the gender. Therefore, Study 2 used voice to manipulate the gender of service robots. Third, Study 2 tested the mediating effect of performance expectancy and trust.

##### Pretest

The gender stereotypes of hotel bellmen were examined in the pretest. A total of 60 participants (53.3% Female; *M*_age_ = 26.73, SD = 2.95) were asked to report their gender stereotypes about this position (same items as Study 1). The result of a *T*-test showed that the gender stereotypes perception of the hotel bellmen was significantly higher than median 4 (*M*_Stereotype_ = 5.58, *t*_(59)_ = 13.246, *p* < 0.001), confirming the male hotel bellmen.

We also tested the experimental materials in the pretest. There were 62 participants (48.4% Female; *M*_age_ = 27.08, SD = 2.88) who were randomly assigned to two groups. Next, participants respectively listened to the same voice message read by a male or female: “Welcome to X wisdom hotel! I'm your bellman. It's my pleasure to serve you!”. Then participants were asked to answer the question: “Do you think the intelligent bellman service robot is male or female?” The results showed that in the female service robot group, thirty participants correctly identified gender, and one participant incorrectly identified gender. In the male service robot group, 28 participants correctly identified gender, and three participants incorrectly identified gender. The Chi-square test showed significant gender differences between the groups (*p* < 0.001).

##### Procedure and participants

Study 2 used a single-factor (Stereotypes: consistency vs. inconsistency) between-subject design. A total of 86 participants (52.3% Female; *M*_age_ = 26.17, SD = 2.94) were randomly assigned to two groups (Stereotypes: consistency vs. inconsistency). First, participants were required to read the following message: “Imagine that you are going on holiday and carrying heavy luggage. When you arrive at the gate of the hotel, an intelligent bellman service robot says ‘welcome to X wisdom hotel! I'm your bellman. It's my pleasure to serve you!' to you.” For each group, there was one of the two voice messages which had no difference except the voice of male or female. After listening to the voice message, participants completed the questionnaire. The questionnaire consisted of three parts. The first part measured the occupational gender stereotypes perception of service robots and willingness to use service robots as Study 1. In the second part, we measured the performance expectancy and trust of the service robot. The performance expectancy was measured by three items adapted from Venkatesh et al. (2003): “This intelligent bellman service robot is useful in the hotel service”; “This intelligent bellman service robot can accomplish check-in quickly”; “Using this intelligent bellman service robot increases my check-in efficiency.” Trust was measured with four items adapted from Siguaw et al. (1998) and Flavian et al. (2006): “I think this intelligent bellman service robot has the necessary capabilities to solve problems encountered in the service,” “I think this intelligent bellman service robot has enough experience to solve the problems encountered in the service,” “I think this intelligent bellman service robot has the necessary resources to solve the problems encountered in the service,” “I think the big data behind this intelligent bellman service robot understands the problems that customers have and can provide them with the services they need” (all measures were rated on a 7-point scale, 1 = Strongly disagree, 7 = Strongly agree). In the third part, participants reported their demographic information.

#### Results

##### Manipulation check

The result of the *T*-test demonstrated that the gender stereotypes perception of the hotel bellman was significantly higher than the median 4 (*M*_Stereotype_ = 5.66, *t*_(85)_ = 19.864, *p* < 0.001). In the consistency group, 41 participants correctly identified the service robot as male and two participants incorrectly identified the service robot as female. Similarly, in the inconsistency group, 39 participants correctly identified the service robot as female and four participants incorrectly identified the service robot as male. After deleting those failing to judge the gender of service robot correctly, 80 samples (*N*_consistency_ = 41, *N*_inconsistency_ = 39; 52.5% Female; *M*_age_ = 26.55, SD = 2.97) were retained. Overall, the manipulation of experience was successful.

##### Dependent variable

One-way ANOVA showed that the willingness to use the service robot (α = 0.822) was significantly higher in the consistency group than in the inconsistency group (*M*_consistency_ = 5.97, SD = 0.69; *M*_inconsistency_ = 5.40, SD = 0.49; *F*_(1, 78)_ = 18.22, *p* < 0.001, η^2^ = 0.358). Thus, H1 was supported.

##### Mediating effect

One-way ANOVA showed that the difference between groups in terms of performance expectancy (*M*_consistency_ = 5.81, SD = 0.42; *M*_inconsistency_ = 5.23, SD = 0.53; *F*_(1, 78)_ = 28.88, *p* < 0.001, η^2^ = 0.270) and trust (*M*_consistency_ = 5.63, SD = 0.45; *M*_inconsistency_ = 5.02, SD = 0.38; *F*_(1, 78)_ = 43.20, *p* < 0.001, η^2^ = 0.356) was significant. A bootstrap method was used to test the mediating effect of performance expectancy and trust, with a 95% bias-corrected bootstrap (Model 4 in PROCESS) based on 5,000-sample confidence intervals (CI). The results showed that the mediating effect of performance expectancy [β = 0.283, 95% CI = (0.164, 0.418), excluding 0] and trust [β = 0.362, 95% CI = (0.167, 0.564), excluding 0] were significant. After controlling the mediators, the consistency between occupational gender stereotypes and gender of the service robot on willingness to use the service robot was not significant [β = −0.074, 95% CI = (−0.350, 0.201), including 0]. Thus, performance expectancy and trust together constitute full mediators, and H2 and H3 were supported.

#### Discussion

Study 2 replaced a position with male occupational stereotypes and tested the assumptions with a voice manipulated to sound like the assigned gender of the service robots. Again, these experimental results prove that the consistency between occupation gender stereotypes and service robot gender can improve the customer's willingness to use the robot. We enhanced the robustness of the results by changing the experimental scenario and materials. In addition, Study 2 proved that performance expectancy and trust fully mediated the effect of occupational gender stereotypes consistency on customers' willingness to use service robots.

### Study 3

#### Research design

Study 3 explored a boundary condition for the effect of the consistency between occupation gender stereotypes and service robots' gender on the customers' willingness to use service robots. The experiment entailed a 2 (Stereotype: consistency vs. inconsistency) × 2 (service context: service success vs. service failure) between-subjects design. We used an occupation scenario of a hotel receptionist and experimental materials from Study 1.

##### Procedure and participants

One hundred and seventy-six participants (51.1% Female; *M*_age_ = 26.41, SD = 2.93) were randomly assigned to four groups. Participants were first required to read the following message: imagine that you check in at a self-service hotel on holiday. You ask the hotel receptionist service robot “Please recommend some local cuisine.” The gender of the service robot is manipulated by the visual image, the same as Figure 2 of Study 1. For the service success group, the service robot showed a list of the top 10 local food restaurants; for the service failure group, the service robot does not follow you “Sorry, I don't understand. Could you say it again?” The participants then completed the questionnaire and reported their demographic information. The questionnaire consisted of two parts. In the first part, we measured the occupational gender stereotypes perception, the willingness to use service robots, performance expectancy, and trust in service robots (same items as Study 2). In the second part, participants reported their demographic information.

#### Results

##### Manipulation check

Single sample *T*-test demonstrated that the occupational gender stereotype perception score of the hotel receptionist service robot was significantly lower than the median value of 4 (*M*_Stereotype_ = 2.78, *t*_(175)_ = −27.595, *p* < 0.001). In the consistency group, 86 participants correctly identified the gender of the service robot as female and two participants incorrectly identified the gender of the service robot as male. In the inconsistency group, 82 participants correctly identified the gender of the service robot as male and four participants incorrectly identified the gender of the service robot as female. After deleting those failing to judge the gender of service robot samples, 168 samples (*N*_consistency_ = 84, *N*_inconsistency_ = 84; 52.4% Female; *M*_age_ = 26.33, SD = 2.89) were retained. Thus, the manipulation of experience was successful.

##### Dependent variable

**Figure 3:**
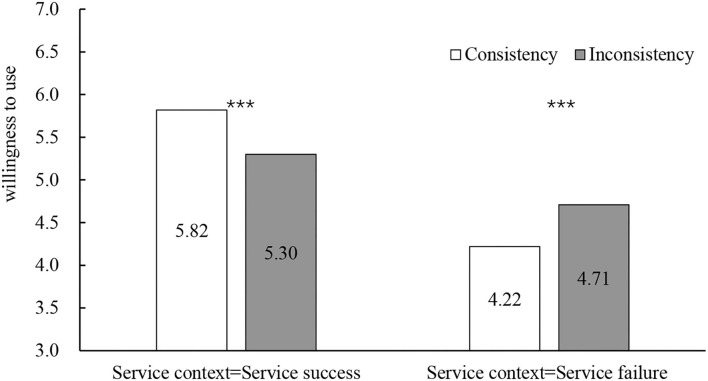
The influence of occupational gender stereotypes on willingness to use. *p < 0.05, **p < 0.01, ***p < 0.001.

Two-way ANOVA showed that, for willingness to use (see Figure 3), the main effect of consistency was not significant (*M*_consistency_ = 5.02, SD = 0.93; *M*_inconsistency_ = 5.00, SD = 0.51; *F*_(1, 164)_ = 0.030, *p* = *0.862*, η^2^ = 0.000), the main effect of service failure was significant (*M*_success_ = 5.56, SD = 0.52; *M*_failure_ = 4.46, SD = 0.50; *F*_(1, 164)_ = 265.43, *p* < 0.001, η^2^ = 0.612), the interaction effect between the consistency of service robot's occupation stereotypes and service failure was significant (*F*_(1, 164)_ = 56.89, *p* < 0.001). In service success scenario, the consistency between gender and occupation stereotypes had a significant positive effect on willingness to use the service robot (*M*_consistency_ = 5.82, SD = 0.48; *M*_inconsistency_ = 5.30, SD = 0.42; *F*_(1, 82)_ = 28.46, *p* < 0.001, η^2^ = 0.204). In service failure scenario, the consistency between service robot's gender and occupation stereotypes had a significant negative effect on willingness to use the service robot (*M*_consistency_ = 4.22, SD = 0.44; *M*_inconsistency_ = 4.71, SD = 0.42; *F*_(1, 82)_ = 28.45, *p* < 0.001, η^2^ = 0.186).

**Figure 4:**
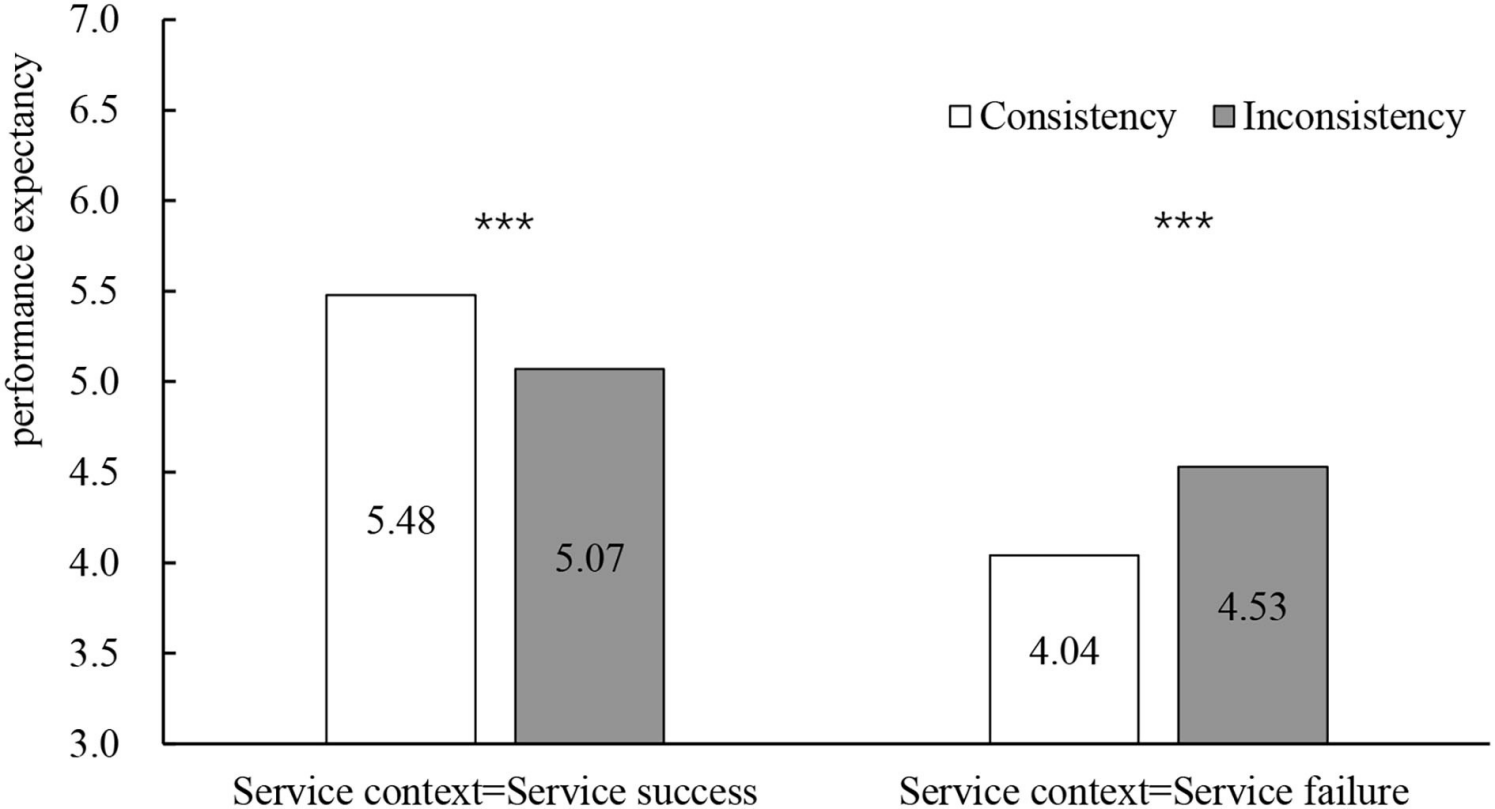
The influence of occupational gender stereotypes on performance expectancy. *p < 0.05, **p < 0.01, ***p < 0.001.

**Figure 5:**
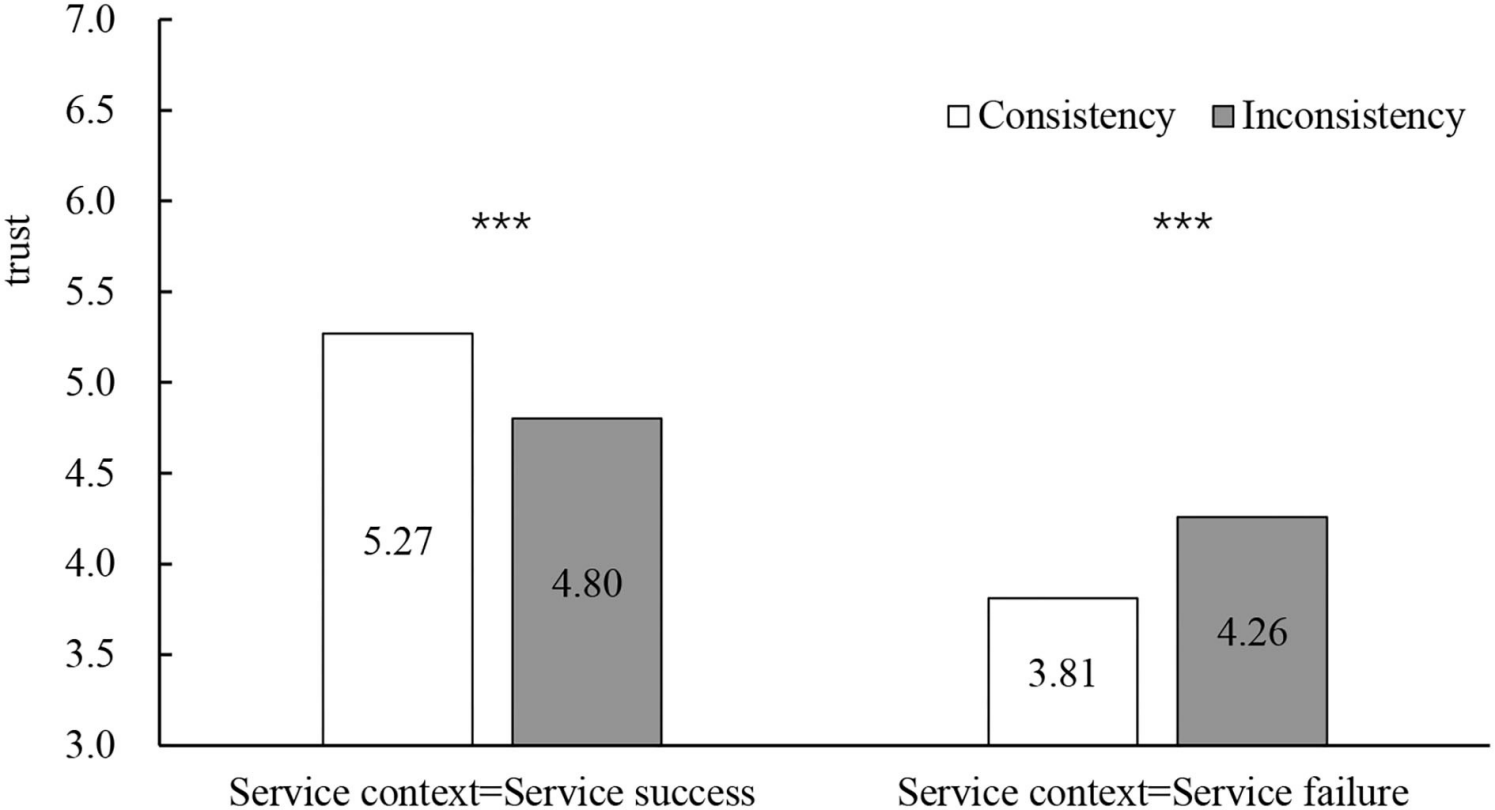
The influence of occupational gender stereotypes on trust. *p < 0.05, **p < 0.01, ***p < 0.001.

In the service success scenario, the consistency between gender and occupation stereotypes had a significant positive effect on performance expectancy (*M*_consistency_ = 5.48, SD = 0.48; *M*_inconsistency_ = 5.07, SD = 0.50; *F*_(1, 82)_ = 15.07, *p* < 0.001, η^2^ = 0.240) and trust (*M*_consistency_ = 5.27, SD = 0.56; *M*_inconsistency_ = 4.80, SD = 0.64; *F*_(1, 82)_ = 13.17, *p* < 0.001, η^2^ = 0.362). In the service failure scenario, the consistency between gender and occupation stereotypes had a significant negative effect on performance expectancy (*M*_consistency_ = 4.04, SD = 0.50; *M*_inconsistency_ = 4.53, SD = 0.42; *F*_(1, 82)_ = 23.65, *p* < 0.001, η^2^ = 0.215) and trust (*M*_consistency_ = 3.81, SD = 0.52; *M*_inconsistency_ = 4.26, SD = 0.40; *F*_(1, 82)_ = 19.51, *p* < 0.001, η^2^ = 0.215; see Figures 4, 5).

##### Moderated mediating effect

A bootstrap method (PROCESS, Model 7, 5,000 samples, 95% bias-corrected confidence) was conducted to test the moderated mediating effect of performance expectancy and trust. Taking the consistency of occupation gender stereotypes as the independent variable, performance expectancy and trust as the mediator, service failure as the moderator, and willingness to use as the dependent variable to test. For willingness to use, the index for the full model was significant [performance expectancy: moderated mediation index = −0.462, 95% CI = (−0.717, −0.250), not included 0; trust: moderated mediation index = −0.339, 95% CI = (−0.561, −0.147), not included 0].

In the service success scenario, the mediating effects of performance expectancy [β = 0.211, 95% CI = (0.093, 0.365), not included 0] and trust [β = 0.175, 95% CI = (0.060, 0.318), not included 0] were significant, and the mediating effects were positive. In the service failure scenario, the mediating effects of performance expectancy [β = −0.251, 95% CI = (−0.411, −0.122), not included 0] and trust [β = −0.164, 95% CI = (−0.284, −0.064), not included 0] were significant, yet the mediating effects were negative. After controlling the mediating effect, the direct effect of the consistency between service robot's gender and occupation stereotypes was no longer significant [β = 0.026, 95% CI = (−0.093, 0.145), included 0], which showed that performance expectancy and trust played a fully mediating role together. Thus, H4 and H5 were supported.

#### Discussion

In the hospitality industry, service failure is a very common situation and significantly impacts customer satisfaction and willingness to use the service again. Therefore, Study 3 explored a service failure as the boundary condition for the effect of the consistency between occupation gender stereotypes and service robot gender on the customers' willingness to use service robots. The results indicate that when a service failure happens, the consistency between occupation gender stereotypes and the service robot's gender decreases customers' willingness to use the service robot. In service failure, when the service robot's gender is consistent with occupation gender stereotypes, customers will have a lower performance expectancy and trust for a service robot. Customers expect service robots consistent with occupation gender stereotypes to behave better, so when a service failure happens, customers will feel less satisfied and less willing to use the service robot. When occupational gender stereotypes are inconsistent with the service robot's gender, customers' tolerance of service failure will increase because they think this service robot is not suitable for this position. Study 3 explored and tested a boundary condition for the effect by changing the experimental scenario.

## Discussion and conclusion

Occupational gender stereotype is a common social phenomenon (White and White, 2006; Haines et al., 2016). In the service context of the hospitality industry, customers activate occupational stereotypes when facing service providers in different specific positions. With the wide application of AI technologies in hotels, service robots, which gender-neutral entities, are gradually being imbued with gender characteristics. How to appropriately apply service robots with obvious gender characteristics into service processes is the key to the successful application of service robots on a wide scale. That is also the focus of the current research. Three studies were conducted to examine the effect of consistency between occupational gender stereotypes and service robot gender on customers' willingness to use, its underlying psychological mechanism, and the boundary condition. The findings are as follows:

First, the consistency between occupational gender stereotypes and service robot gender significantly influences the willingness to use service robots. Specifically, consistency can improve customers' willingness to use it compared to inconsistency, which illustrates that gender is an important factor impacting the acceptance of service robots.

Second, performance expectancy and trust are the underlying psychological mechanisms and play mediating roles in the above relationship. When it comes to the consistency between occupational gender stereotypes and service robot gender, customers think that service robots have the competency to meet the occupational demands and solve problems within their assigned work scope, and thus produce higher performance expectancy, enhancing willingness to use. In a similar vein, customers have faith that these robots can serve them well due to their competency and are also therefore more likely to trust service robots, which in turn increases their willingness to use the mechanical service providers. Contrarily, when the occupational gender stereotypes are inconsistent with the gender of service robots, customers suspect service robots' ability is mismatched with their job requirements and therefore it is incapable of completing the task. As a result, customers' performance expectancy and trust are damaged and their willingness to use is reduced.

Third, we find that service failure is a boundary condition. When service failure happens, the consistency between occupational gender stereotypes and service robot gender reduces customers' willingness to use. The reason is that customers tend to have higher performance expectancy and trust toward the gendered service robots who keep consistent with their occupational gender stereotypes. Once service goes wrong, service robots fail to live up to customers' expectations and trust, and customers feel more disappointed.

### Theoretical implications

First, our research reveals that customers' willingness to use service robots is affected by the consistency between the gender of the robots and corresponding occupational gender stereotypes, which broadens the literature on the technology acceptance model (TAM) in the service context. Although prior research based on the AIDUA model has explored the impact of anthropomorphism on traditional self-service technology and emergent smart technologies (Destephe et al., 2015; Wirtz et al., 2018), few studies have taken a closer look at the facets of anthropomorphism. Our research is grounded on social role theory to examine and suggest that the gender of service robots, as one key facet of anthropomorphism, consistently combined with occupational stereotypes can improve customers' usage intention. By doing so, we improve the understanding of customers' acceptance and responses to technology usage, especially service robot usage in service settings, thereby expanding the research on the antecedents of TAM in terms of anthropomorphism.

Second, our research suggests that the positive effect of occupational gender stereotypes consistency remains in the human-robot service interaction, extending the scope of relevant literature. Specifically, previous literature has confirmed the benefits of that consistency mainly concentrating on human interactions (Tay et al., 2014), but has not proven whether the findings apply to human-robot interaction, or whether it further improves the willingness of customers to use service robots. Therefore, we add to the literature on occupational gender stereotypes from the perspective that robot gender can also trigger gender stereotypes and produce similar consequences if kept consistent.

Finally, we reveal the psychological mechanisms that explain the positive influence gendered service robots' have on customers' acceptance when they are consistent with specific service positions, which helps open the “black box” of the positive effect of gender stereotype consistency. Many previous studies have proven that occupational gender stereotypes and service providers' gender consistency bring benefits, but few delve into the reasons for this (Mohr and Henson, 1996; Pinar et al., 2017). Rooted in the field of human-robot interaction, this study verifies the mediating role of performance expectation and trust, deepening the understanding of gender stereotypes.

### Managerial implications

Findings from this research can provide practical implications in three ways:

First, hotels should clearly identify service positions that involve specific occupational gender stereotypes since it lays the foundation for designing and deploying anthropomorphic service robots. Specifically, hotels can collect and analyze the gender stereotypes of each service position through surveys or interviews with customers, and then find out which positions are more masculine, and which are more feminine in the eyes of customers. The next step is to deploy service robots whose genders positively correspond to these positions. In terms of manipulations of robot gender, relevant studies have proven that elements such as hairstyle, voice, body shape, and names contribute to differentiating the gender of robots (Eyssel and Hegel, 2012; Nomura, 2017; Ahn et al., 2022). Therefore, hotels can introduce gendered robots based on these features, and introduce them into positions that match their genders and meanwhile conform to customers' stereotypes. By doing so, we expect customers' willingness to use service robots to be increased markedly.

Second, since the effect of robot gender and occupational gender stereotypes consistency on consumers' willingness to use them depends on performance expectancy and trust of customers, we suggest two ways that hotels can do this. For one thing, hotels can design service robots to actively show competency in solving problems and satisfying customers' needs, in hope of improving customers' performance expectancy for robots. For example, hotels can leverage online or offline advertising to highlight the robots' accuracy, reliability, and efficiency (Gursoy et al., 2019). Once customers touch the robots, robots can also introduce themselves by claiming what they are capable of with a female or male voice, which also helps improve the performance expectancy of customers and further improve willingness to use. For another, an emphasis on the ability of robots also applies to trust - the other critical determinant. In addition, hotel managers should clarify that robots respect customers' privacy, and never leak their personal information to others, which is helpful in enhancing customers' trust, thereby improving customers' willingness to use service robots.

Finally, we remind hotel managers to be extraordinarily cautious when robots fail to serve customers well because according to our findings, the consistency between service robot gender and occupational gender stereotypes in service failures damages customers' willingness to use robotic service providers. To deal with this, managers and robot designers can make robots automatically initiate an apology or explanation to customers and ask for recovery when service failure happens (Choi et al., 2021). When necessary, human intervention may be feasible. After all, human employees are more flexible to deal with customers than humanoid robots.

### Limitations and future research directions

Our research still has several limitations and is worthy of future exploration. First, the hospitality industries involve hotels, restaurants, and scenic spots. This research only selected hotels as the context to explore the hypotheses. Whether our findings hold in other human-robot interaction contexts needs to be further tested. Second, this research focuses on the gender of service robots to discuss occupational stereotypes, but in practice, there are other humanoid elements being applied to anthropomorphize service robots, such as cuteness, emotionality, and social interactivity. Future scholars can examine if there is any conflict between gender characteristics with these humanoid elements which may backfire and damage the acceptance of service robots. Third, this paper used scenario-based experiments to confirm the effect of consistency on the customers' willingness to use and the underlying mechanisms. Scenario experiments have the advantage of high internal validity, but there are still some differences with real service contexts. Future research can confirm our findings with the field study. Finally, although occupational gender stereotypes are hard to change in the short term, to build a better society, it is of great value to find out the methods to break gender stereotypes in service contexts, regardless of service robots or human employees.

## Data availability statement

The raw data supporting the conclusions of this article will be made available by the authors, without undue reservation.

## Ethics statement

Ethical review and approval was not required for the study on human participants in accordance with the local legislation and institutional requirements. Written informed consent from the patients/ participants or patients/participants legal guardian/next of kin was not required to participate in this study in accordance with the national legislation and the institutional requirements.

## Author contributions

QH contributes to the theoretical building of the paper and wrote the initial draft. XP conceived the idea, collected the data, and analyzed the data. JL additional literatures and made further editing on the paper. YY collected the data and takes responsible for paper submission. All authors discussed the structure of the paper and finalized the manuscript.

## Conflict of interest

The authors declare that the research was conducted in the absence of any commercial or financial relationships that could be construed as a potential conflict of interest.

## Publisher's note

All claims expressed in this article are solely those of the authors and do not necessarily represent those of their affiliated organizations, or those of the publisher, the editors and the reviewers. Any product that may be evaluated in this article, or claim that may be made by its manufacturer, is not guaranteed or endorsed by the publisher.

## Figures and Tables

**Figure:**
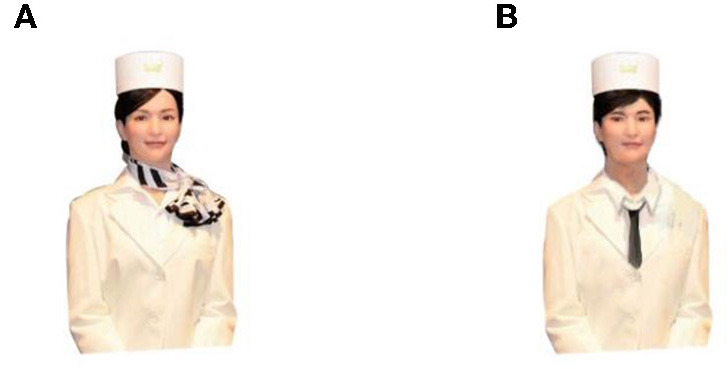
**(A)** Female hotel receptionist service robot. **(B)** Male hotel receptionist service robot.

## Appendix

### Appendix 2

Suppose you are on a vacation and are going to a reserved unmanned hotel. When you come to the hotel to check in, the reception intelligent robot first greets you: Consistency: Hello, welcome to the hotel! I am the reception intelligent robot Meiling. Your check-in process will be handled by me. If you have any questions or requirements during your stay, you can give me feedback! Thank you for choosing X hotel, I hope you have a wonderful day! Inconsistency: Hello, welcome to the hotel! I am the reception intelligent robot Jiahao. Your check-in process will be handled by me. If you have any questions or requirements during your stay, you can give me feedback! Thank you for choosing X hotel, I hope you have a wonderful day!
